# Beyond Bioenergetics: Moonlighting Functions of F_1_F_O_‐ATPase From ATP Synthesis to an ATP‐Wasting Death Switch

**DOI:** 10.1002/med.70088

**Published:** 2026-07-13

**Authors:** Salvatore Nesci

**Affiliations:** ^1^ Department of Veterinary Medical Sciences University of Bologna Ozzano Emilia Italy

**Keywords:** cation cofactor switching, F_1_F_O_‐ATPase, mitochondrial permeability transition pore, regulated cell death

## Abstract

Mitochondrial F_1_F_O_‐ATPase is classically viewed as the “splendid” rotary nanomachine that sustains life by converting the proton‐motive force (pmf) into ATP. Yet this same complex can adopt a second, context‐dependent function that links bioenergetics to cell fate. When oxygen becomes limiting during ischemia, stroke, hypoxia, or anoxia, respiratory chain activity declines, pmf collapses, and the enzyme reverses direction: ATP hydrolysis drives rotor turnover and pumps H^+^ across the inner mitochondrial membrane to partially restore pmf. This reverse mode can be protective by preserving membrane potential and basic mitochondrial homeostasis, but it also imposes a severe energetic burden on the cell. A key determinant of whether F_1_F_O_‐ATPase acts as an energy‐conserving or energy‐dissipating machine is the identity of the catalytic divalent cation. Under physiological conditions, Mg^2+^ supports efficient, reversible ATP synthesis/hydrolysis. In pathology, mitochondrial Ca^2+^ overload may replace Mg^2+^ at catalytic sites, shifting catalysis toward ATP hydrolysis and promoting an ATP‐wasting state that triggers mitochondrial permeability transition pore (mPTP) formation and regulated cell death. Thus, ATP hydrolysis by F_1_F_O_‐ATPase beyond regulation by pmf and inhibitory proteins such as IF1, cation cofactor selection emerges as a decisive switch that repurposes the enzyme from “enzyme of life” to “enzyme of death.” Here, this cofactor‐dependent reversibility is framed as a moonlighting role of F_1_F_O_‐ATPase, integrating energy conversion with death signaling. Conceptual questions are outlined to resolve current debate and to exploit this switch for therapeutic insight.

Defined as a “splendid molecular machine” [1], the F_1_F_O_‐ATPase (ATP synthase) is a ubiquitous rotary enzyme central to bioenergetics in mitochondria and conserved in chloroplasts and bacteria [2]. Its effectiveness comes from coupling chemistry to mechanics across two domains. The membrane‐extrinsic F_1_ domain is the catalytic head, where nucleotides bind, and ATP catalysis occurs. The membrane‐intrinsic F_O_ domain is embedded in the inner mitochondrial membrane (IMM) and converts the proton‐motive force (pmf), the electrochemical gradient of protons generated by the respiratory chain, into mechanical rotation. A rotating central stalk links the F_O_ rotor to the (αβ)_3_ catalytic head. As H^+^ flow down the pmf through F_O_, the rotor turn, driving cyclical conformational changes of the three β subunits that bind ADP and phosphate, synthesize ATP, and release product [3]. Under aerobic conditions, this is the enzyme of life, delivering most cellular ATP. Yet the same architecture is fully reversible. When the pmf is low, and glycolytic ATP is available, ATP hydrolysis in F_1_ can drive rotation in the opposite direction and pump H^+^ back across the IMM, partially rebuilding pmf [4].

During hypoxia, anoxia, or ischemia, mitochondrial electron transport slows or stops, the pmf decreases, and mitochondria risk losing membrane energization required for ion homeostasis, metabolite exchange, protein import, and Ca^2+^ handling. Reverse catalysis of F_1_F_O_‐ATPase can act as an emergency response, consuming ATP to generate pmf long enough for mitochondrial functionality. But this rescue plan is energetically expensive. The chemical potential released by the negative Gibbs free energy of ATP hydrolysis, which the enzyme exploits to re‐energize the IMM, can rapidly deplete the cell of ATP needed elsewhere. If prolonged, reversal turns mitochondria into ATP consumers, undermining metabolic efficiency and accelerating bioenergetic failure. F_1_F_O_‐ATPase therefore, restrains the reverse mode with regulated processes pH‐dependent. When pmf falls and ATP is excessively hydrolyzed inducing cellular acidification, mitochondria can engage Inhibitor Factor 1 (IF1). This protein binds the F_1_ catalytic head and selectively inhibits ATP hydrolysis without blocking ATP synthesis [5]. This directionality matters because synthesis corresponds to the opposite direction of rotation. IF1 activity is tuned by physiological context, including pH and oligomerization. Mechanistically, IF1 first engages the most open catalytic conformation (the empty β_E_ site) and then stabilizes an inhibited state after a small number of ATP‐driven rotational steps, thereby placing a hard limit on ATP wastage when respiratory ATP production fails [6].

This pmf versus IF1 logic emphasizes an often‐overlooked principle. In mitochondria, pmf generated by respiration or by ATP hydrolysis functions as a primary bioenergetic event that maintains mitochondrial integrity and function. Maintaining an energized IMM is the immediate prerequisite for many mitochondrial processes. Even with pmf and IF1, a deeper question remains. Can the cation cofactor itself rewire what F_1_F_O_‐ATPase does?

Under physiological conditions, Mg^2+^ occupies the canonical catalytic binding site and supports efficient, cooperative, reversible catalysis [7]. Under pathological conditions, mitochondria can accumulate large amounts of Ca^2+^. Massive and persistent cytosolic Ca^2+^ increase is drawn into the matrix by the strong negative membrane potential. Ca^2+^ has an uncompetitive mechanism with Mg^2+^ binding catalytic sites [8]. Because Ca^2+^ is larger radius than Mg^2+^, it reshapes coordination chemistry at the metal‐nucleotide complex. Mg^2+^ commonly adopts a relatively rigid octahedral arrangement, whereas Ca^2+^ reaches higher and more irregular coordination numbers. Substituting Ca^2+^ for Mg^2+^ is therefore expected to distort active‐site geometry, lose catalytic cooperativity among β subunits, and shift the balance of mechanochemical coupling toward a less efficient, less regulated form of ATP hydrolysis [9] (Figure 1).

**Figure 1:**
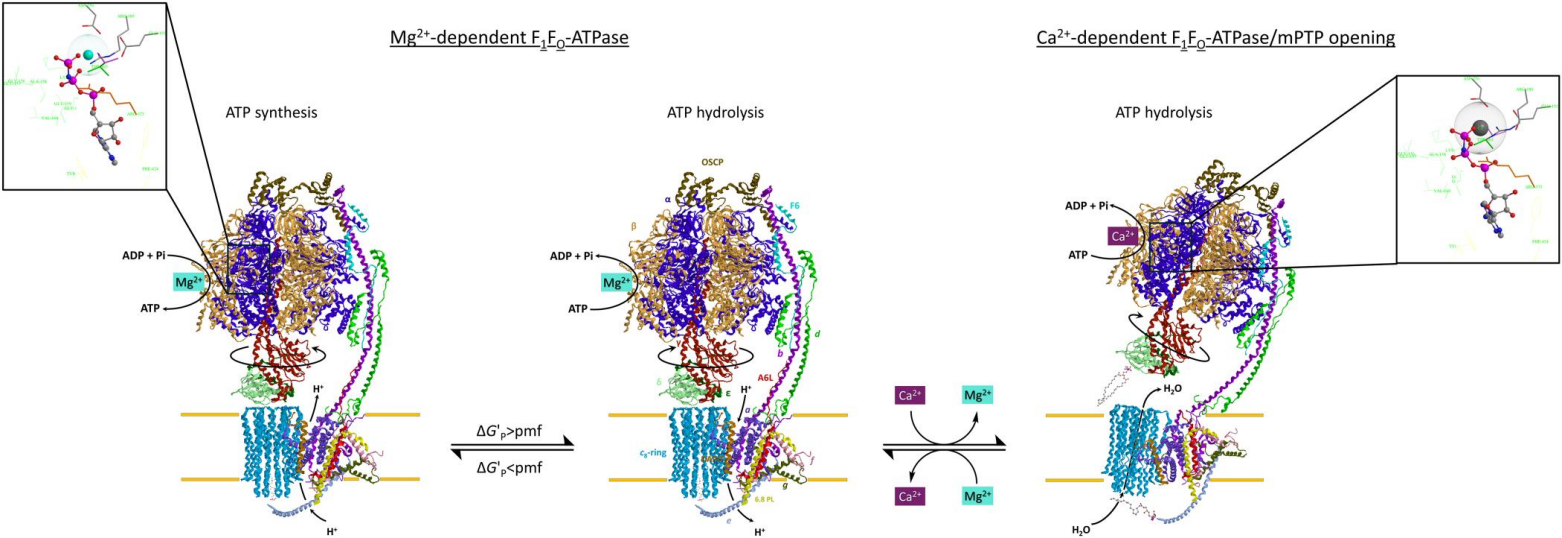
Structure of F_1_F_O_‐ATPase of mammalian mitochondria and functional duality. The enzyme subunits are drawn as ribbon representations obtained from the modified PDB ID code: 6TT7. F_1_F_O_‐ATPase synthesis or hydrolysis are raised by Mg^2+^ or ATP hydrolysis by Ca^2+^ as cofactors coupled to H^+^ translocation. Ca^2+^‐dependent F_1_F_O_‐ATPase suggests model of the PTP opening with irreversible dissociation of the F_1_ domain from F_O_ domain during prolonged openings of the c‐ring channel under pathological conditions. In the boxes, the ground‐state structures of the catalytic subunit show distinct conformations in the β_DP_ subunit depending on whether Mg^2+^ or Ca^2+^ is bound (PDB ID: 2JDI), which depict the interactions of Mg‐ATP (Mg^2+^ shown as a turquoise sphere) and Ca‐ATP (Ca^2+^ shown as a purple sphere), respectively. Key β_DP_ residues involved in ATP binding in the presence of Mg^2+^ (on the left) or Ca^2+^ (on the right) are indicated, and the electron density for Mg^2+^ and Ca^2+^ is rendered as a sphere. The aminoacids of P‐loop are in green, and side chains are shown as wireframe. The β_Thr163_ side chain (pink stick) makes a direct electrostatic interaction with the divalent cation, while β_Arg189_, β_Glu192_, and β_Asp256_ complete cation coordination via three ordered water molecules (not shown). The arginine finger (α_Arg373_; orange stick) helps position the γ‐phosphate correctly in the binding pocket. Aminoacid acronyms and the position interacting with ATP and the cation cofactor are shown in green. The letter colors are the same as those of the subunits to which the structures belong. Δ*G*'_P_, Gibbs free energy of ATP hydrolysis; pmf, proton‐motive force. [Color figure can be viewed at wileyonlinelibrary.com]

Functional observations on Ca^2+^‐dependent ATP hydrolysis indicate that F_1_F_O_‐ATPase can still generate torque linked to F_O_ function of H^+^ pump [9, 10]. The ATP hydrolysis is inhibited by canonical F_1_ inhibitors and oligomycin‐sensitive, consistent with dependence on the F_O_ H^+^ pathway and rotor mechanics. In inside‐out submitochondrial particles, a respiration‐generated pmf can oppose Ca^2+^‐dependent H^+^‐translocation F‐ATPase activity, whereas protonophores that collapse pmf can stimulate ATP hydrolysis [9]. These findings are consistent with a rotary motor of ATP hydrolysis coupled to an H^+^ pump that can still operate with Ca^2+^, even if the net bioenergetic return is poor.

A particularly intriguing implication is that Ca^2+^ may alter the functional role of F_1_F_O_‐ATPase. Recently, Ca^2+^‐dependent F_1_F_O_‐ATPase has been linked to opening of the mitochondrial permeability transition pore (mPTP) [11, 12], a large non‐selective conductance that permeabilizes the IMM to solutes up to roughly 1.5 kDa. mPTP opening collapses the membrane potential, disrupts osmotic balance, promotes swelling, and can trigger regulated cell death pathways. In the integrative “moonlighting” framework proposed here, building on prior work on the Ca^2+^‐F_1_F_O_‐ATPase/mPTP axis, a normally energy‐generating enzyme is interpreted as functionally repurposed under pathological Ca^2+^ overload, shifting toward inefficient ATP hydrolysis, impaired pmf maintenance, and permeability‐transition‐associated cell‐death signaling.

A plausible mechanistic route is structural plasticity. Ca^2+^‐driven changes at catalytic sites could propagate through the (αβ)_3_ head into the peripheral stalk and toward the F_O_ rotor–stator interface, reshaping how mechanical strain is distributed across the complex [12]. Even if ATP‐driven rotation persists, the net energetic outcome may be dominated by leak: weak net pmf generation during Ca^2+^‐dependent turnover could reflect H^+^ backflow through a pore‐like state rather than a complete uncoupling of rotation from H^+^ translocation. In other words, the motor may continue to rotate to induce the conformational change that drives mPTP formation (Figure 1), and the membrane may no longer maintain the pmf.

Comparative biology suggests that the capacity for such switching is embedded rather than accidental. The modular architecture of F_1_ catalytic head, coupled to F_O_ H^+^ translocator and the rotary chemomechanical mechanism are conserved from invertebrates to vertebrates [13]. While the energetic cost of ATP synthesis varies with c‐ring stoichiometry [13], the core engine is ancient, and associations between Ca^2+^‐supported activity and permeability transition have also been reported beyond mammals.

At the same time, the field remains divided on whether F_1_F_O_‐ATPase is itself the pore, a core component of it, or a regulator of a pore formed by other proteins and lipids. That debate is healthy because permeability transition is shaped by membrane environment, redox state, and protein‐protein interactions, and mitochondria contain multiple stress‐activated conductances. What is increasingly hard to ignore is the experimental opportunity. Mg^2+^ versus Ca^2+^ cofactor axis provides a concrete chemical lever linking bioenergetic regulation to cell‐fate decisions [14].

On balance, a clear priority is the integration of structural, single‐molecule, and physiological experiments that track divalent‐cation occupancy, catalytic intermediates, rotor dynamics, IF1 regulation, and membrane permeability in the same systems, moving from purified complexes to intact mitochondria and in vivo disease models. Resolving how Ca^2+^ converts a reversible energy‐conserving enzyme into an energy‐dissipating death effector could open new strategies for conditions in which mitochondrial Ca^2+^ overload and permeability transition drive tissue injury. Thus, the Mg^2+^/Ca^2+^ control of F_1_F_O_‐ATPase activity provides not only a mechanistic explanation for the transition from energy conservation to permeability‐transition‐associated cell death, but also a pharmacological framework for drug discovery [15]. Therapeutic strategies may include limiting mitochondrial Ca^2+^ overload, desensitizing cyclophilin D‐dependent pore opening, targeting F_1_F_O_‐ATPase OSCP/peripheral‐stalk interfaces, stabilizing Mg^2+^‐supported catalysis, or modulating IF1‐dependent restraint of ATP hydrolysis. These approaches should be prioritized in disease contexts where Ca^2+^ overload, ATP depletion, and mPTP opening are central pathogenic events.

## Conflicts of Interest

The author declares no conflicts of interest.

## Data Availability

Data sharing is not applicable to this article as no data sets were generated or analyzed during the current study.
